# Species turnover and functional nestedness constitute the geographic patterns of stream diatoms in the Three Parallel Rivers region, China

**DOI:** 10.1002/ece3.70010

**Published:** 2024-07-14

**Authors:** Jiancheng Hu, Nuo Xu, Sicheng Ao, Lu Tan, Xianfu Li, Qinghua Cai, Tao Tang

**Affiliations:** ^1^ Institute of Hydrobiology, Chinese Academy of Sciences Wuhan China; ^2^ School of Environmental Science and Engineering Hubei Polytechnic University Huangshi China; ^3^ School of Resources and Environmental Engineering Wuhan University of Technology Wuhan China; ^4^ College of Life Sciences Hubei Normal University Huangshi China; ^5^ Key Laboratory of Urban Environment and Health, Ningbo Observation and Research Station Institute of Urban Environment, Chinese Academy of Sciences Xiamen China; ^6^ Institute of Eastern‐Himalaya Biodiversity Research Dali University Dali Yunnan China

**Keywords:** attached ability, directional spatial processes, dispersal limitation, environmental filtering, functional beta diversity, taxonomic beta diversity

## Abstract

Unraveling biodiversity patterns and their driving processes is paramount in ecology and biogeography. However, there remains a limited understanding regarding the underlying mechanisms of community assembly, particularly in alpine streams where significant elevation gradients and habitat heterogeneity exist. We investigated the patterns and drivers of beta diversity, explicitly focusing on taxonomic and functional diversity, in the three parallel rivers region in China. We employed a beta diversity partitioning approach to examine the turnover and nestedness components of beta diversity and further deconstructed the diatom community into attached and unattached groups. Our results revealed distinct diversity patterns and drivers for taxonomic and functional beta diversity. Specifically, taxonomic beta diversity was mainly driven by the turnover component affected by spatial processes, whereas functional beta diversity was dominated by the nestedness component affected by environmental processes. Furthermore, our analysis of the division of the whole communities demonstrated that the varying responses of benthic diatoms with different attached abilities to environmental filtering, dispersal limitation, and directional flow were the essential reasons for shaping the biodiversity patterns of species turnover and functional nestedness in the alpine stream. Our findings suggested that partitioning beta diversity and dividing the entire community can more deeply infer underlying community assembly processes, thereby providing valuable insights into understanding biodiversity patterns, drivers, and conservation strategies.

## Graphical abstract

This study investigated beta diversity patterns and drivers of taxonomic and functional groups in a global biodiversity hotspot by partitioning beta diversity (into turnover and nestedness components) and dividing the diatom community (attached and unattached groups). Our work revealed distinct diversity patterns and drivers for taxonomic and functional beta diversity, providing valuable insights into understanding biodiversity patterns, drivers, and conservation strategies.

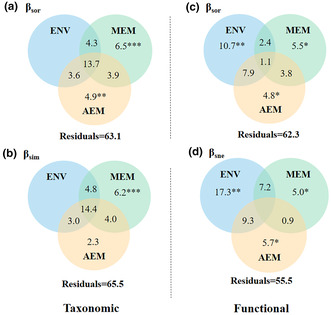

## 1 INTRODUCTION

Beta diversity (β‐diversity) describes variation in metacommunity structure within a study area and has become an effective tool for inferring the spatial patterns in biological communities (Baselga, 2012; Heino, Melo, & Bini, 2015). It connects regional (*γ*) and local (*α*) diversity and better captures the dynamic processes that control community assembly (Heino, Melo, & Bini, 2015; Socolar et al., 2016). However, inferring the change in species composition based solely on the overall beta diversity can be misleading, as the underlying patterns and drivers may be quite different (Baselga, 2010). In recent years, more and more researchers have realized the importance of beta diversity partitioning (Angeler, 2013; Gianuca et al., 2017; Loiseau et al., 2017; Peláez & Pavanelli, 2019). Generally, beta diversity is partitioned into two different processes, namely turnover and nestedness, which describe species replacement and species loss or gain across communities, respectively (Baselga, 2010). These beta diversity components can provide implications for biodiversity conservation. For example, a higher turnover component implies that multiple sites need to be protected, while a higher nestedness component suggests that the richest sites are more important (Angeler, 2013; Peláez & Pavanelli, 2019; Socolar et al., 2016). Furthermore, beta diversity partitioning has been widely used to study the underlying mechanisms of community assembly in various groups, such as birds (Si et al., 2016), trees (Wang et al., 2018), fishes (Peláez & Pavanelli, 2019), macroinvertebrates (Krynak et al., 2019), and phytoplankton (Bohnenberger et al., 2018). However, research on stream benthic diatoms remains limited, considering they always showed multiple metacommunity processes, such as environmental filtering, dispersal limitation, and mass effect (Hu et al., 2022; Wu et al., 2022).

Taxonomic approaches have been widely used to explore the variation in metacommunity composition. However, this approach treats all species as functionally equivalent, thereby overlooking the fact that communities are composed of species with diverse functional strategies (Branco et al., 2020; Loiseau et al., 2017; Siefert et al., 2013). Incorporating functional traits into biodiversity research is essential because functional approaches offer additional insights into biodiversity formation and driving forces (Gianuca et al., 2018; Hill et al., 2019). Although there is a specific correlation between taxonomic and functional diversity, they can have different diversity patterns (Cardoso et al., 2014; Villéger et al., 2013). For example, communities with entirely different species compositions (high taxonomic beta diversity) may be characterized by similar functional traits (low functional beta diversity) (Cardoso et al., 2014). Furthermore, because the effects of environmental processes on metacommunity compositions are mediated by functional traits (e.g., life history, cell sizes, and behavioral characteristics), functional approaches can tell us more about community assembly processes (Heino & Tolonen, 2017; Perez Rocha et al., 2018).

Benthic diatoms, as important primary producers in stream networks, are highly sensitive organisms to local environmental conditions and regional spatial processes (Soininen, 2007; Stevenson et al., 2010; Tang, Niu, & Dudgeon, 2013). They used various functional strategies to adapt to physical interference and environmental pressure (Lange et al., 2016; Passy, 2007). The difference in functional characteristics makes benthic diatoms respond differently to environmental and spatial processes (Lindholm et al., 2018). However, previous studies showed that this differential response to environmental and spatial processes may be masked (Algarte et al., 2014). Several studies have highlighted that the expected responses of diatoms to environmental processes are insensitive in alpine streams, in which strong spatial barriers and directional fast flow may mask environmental influence (Bottin et al., 2014; Dong et al., 2016). The division of the whole community is considered an effective approach according to the functional strategy of organisms (Pandit et al., 2009; Vilmi et al., 2017). Benthic diatoms in the alpine streams resist rushing flow by firmly attaching to the substrate, which is an extremely important adaptive strategy (Algarte et al., 2017). For example, diatoms that can firmly attach to substrata have an environmental competitive advantage under fast flow conditions (Algarte et al., 2017). In contrast, diatoms that loosely attach to substrata may be washed into unsuitable habitats, suggesting a signal of mass effect (Hu et al., 2022; Leboucher et al., 2020). It can be expected that dividing the whole community into different groups based on the attachment strategy of benthic diatoms can provide valuable information for understanding the patterns and drivers of beta diversity and its components in alpine streams.

**FIGURE 1:**
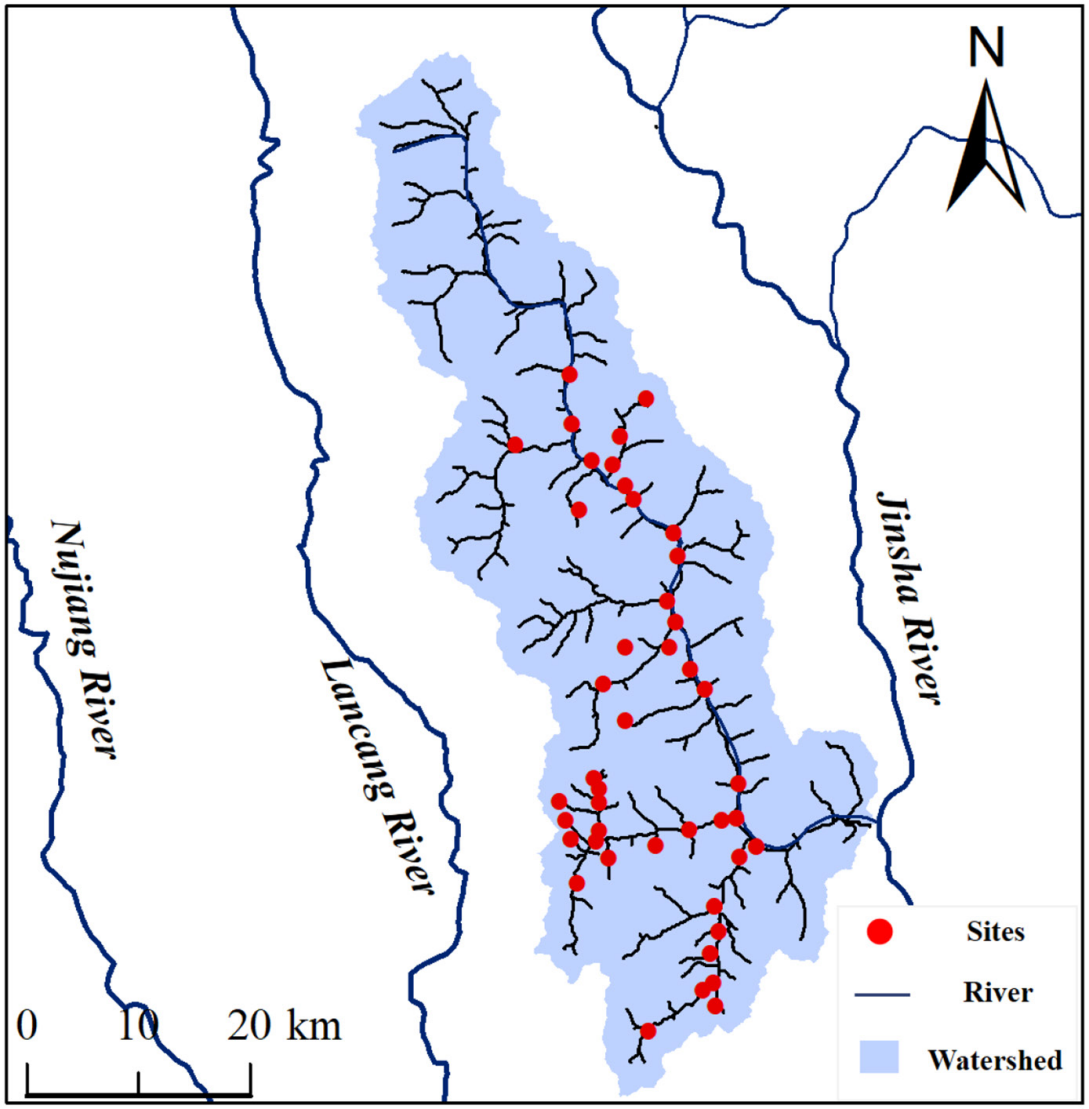
Locations the sampling sites in the Zhubaluo basin of the Three Parallel Rivers region, China.

The Three Parallel Rivers region is located in China and encompasses the confluence of the Jingsha River (Yangtze River), the Lancang River (Mekong River), and the Nu River (Salween River) (Figure 1). This convergence creates diverse geological features and favorable environments for various ecosystems and species. The elevation gradient (ranging from 760 to 6740 m) in the region leads to variations in temperature and precipitation, creating microclimatic conditions that further contribute to the biodiversity. Despite being a global biodiversity hotspot, more information is needed regarding the diversity patterns of diatoms in this region. Here, we investigated beta diversity patterns and drivers of taxonomic and functional groups by partitioning beta diversity (into turnover and nestedness components) and dividing the diatom community (attached and unattached groups). First, we expected that the large elevation gradient and rushing flow in Zhubaluo River would favor high species turnover, while some functional traits would be selective extinction because of environmental filtering caused by habitat heterogeneity (Perez Rocha et al., 2019; Wu et al., 2021). Therefore, we hypothesized that taxonomic beta diversity would exhibit a more pronounced turnover pattern, whereas nestedness components would be dominant for functional beta diversity. Second, because functional traits can better respond to environmental change (Soininen et al., 2016), we hypothesized that functional beta diversity and its components would exhibit greater sensitivity to environmental factors in comparison to taxonomic beta diversity. Third, to gain deeper insights into the underlying mechanisms and determinants of diatom beta diversity, diatom communities were deconstructed based on the attachment ability of the species. We predicted that attached diatom assemblages would be more affected by environmental variables due to their stable habitats, whereas unattached assemblages would be more affected by directional spatial processes because of rushing flow.

## 2 MATERIALS AND METHODS

### 2.1 Study area

Zhubaluo River (27.64–28.09° N, 99.15–99.32° E) is located in the three parallel rivers region (Figure 1). As the tributary on the right bank of Jinsha River, Zhubaluo River origins from Baima Snow Mountain. The river is 104.7 km long, with an elevational gradient of 3127 m, a basin area of 1880 km^2^, and an average annual discharge of 30.2 m^3^/s. The watershed is mainly distributed in the mountain canyons; the land cover is mainly forestland and grassland. We surveyed 44 sample sites in the whole basin in October 2017, and most of the sites are located in the Baima Snow Mountain National Nature Reserve, with a low anthropogenic impact (Figure 1).

### 2.2 Benthic diatoms

The dataset of benthic diatoms was collected by field sampling based on the standard USEPA for national rivers and streams assessment (USEPA, 2007). At each site, 15 benthic substrates were selected from five spots along a 100 m reach to obtain benthic diatom samples. A circular lid (radius: 2.7 cm) was used to confine the sampling area; then, benthic diatoms were brushed into the specimen bottles (Tang et al., 2016). All subsamples from each site were combined into one composite sample and preserved with 4% formalin in the field. In the laboratory, diatoms were identified and counted to species level under a 1000× oil immersion lens after a part diatom sample was acid‐cleaned to make permanent diatom slides. Diatoms were identified following the taxonomic references of Qi (1995), Lange‐Bertalot et al. (2001), Krammer (2000, 2002, 2003), Shi (2004), Hu and Wei (2006), and Li and Qi (2010, 2014). Basionyms of diatoms were examined according to AlgaeBase (https://www.algaebase.org/), and currently accepted names were retained (see Supporting Information).

Benthic diatoms have an essential adaptive strategy that resists physical interference and environmental pressure by attaching to the substrate (Algarte et al., 2017). Based on this attachment strategy, the whole diatom data was divided into attached and unattached diatoms. The attached category comprised species that could firmly attach to stony substrata, whereas the unattached category consisted of species that were loosely attached to the substratum (Heino & Soininen, 2006). In this study, diatom matrices were formed for the attached and unattached categories, consisting of 75 and 39 species, respectively (see Supporting Information).

### 2.3 Functional traits

Thirteen functional traits for benthic diatoms were selected to represent functional compositions that diatoms respond to environmental changes, including three categories: guilds profile, life form, and cell size (Table 1). Guilds profile was proposed by Passy (2007) based on the potential of species to utilize nutrient resources and resist physical interference, including high profile, low profile, and motile profile. Taxa with the same ecological function groups can make use of similar resources, coexist in the same environment, and have different adaptability to the same abiotic factors (Rimet & Bouchez, 2012). Cell size among diatoms is different, which is closely related to nutrient acquisition and distribution (Teittinen et al., 2018). According to the volumes (μm^3^), the cell size was divided into five classes: size1 < 100 ≤ size2 < 300 ≤ size3 < 600 ≤ size4 < 1500 ≤ size5. Diatoms also exhibit a diversity of life forms due to different growth forms (Lange et al., 2016; Rimet & Bouchez, 2012). Referring to the classification information of several papers, diatoms were grouped into five life forms: free move, floating, valve face, stalked, and colonies (Berthon et al., 2011; Law et al., 2014; Marcel et al., 2017; Rimet & Bouchez, 2012). More details on these traits of benthic diatoms in this study can be found in the Supporting Information.

**TABLE 1:** Functional traits used in this study selected for benthic diatoms.

Traits	Trait states	Code	References
Guild profile	Low‐profile	Low	Passy (2007) and Rimet and Bouchez (2012)
	High‐profile	High	
	Motile	Mot	
Cell size	Size < 100 μm^3^	Size1	Rimet and Bouchez (2012) and Teittinen et al. (2018)
	100 ≤ Size < 300 μm^3^	Size2	
	300 ≤ Size < 600 μm^3^	Size3	
	600 ≤ Size < 1500 μm^3^	Size4	
	Size ≥ 1500 μm^3^	Size5	
Life forms	Adnate	Adnate	Berthon et al. (2011), Law et al. (2014), Marcel et al. (2017), and Rimet and Bouchez (2012)
	Colonies	Colonies	
	Floating	Floating	
	Free move	Free move	
	Mucilage	Mucilage	

### 2.4 Environmental and spatial variables

Environmental variables, including physical habitat and water chemistry factors, were monitored at each sample site. Elevation and geographical coordinates were measured by GPS (Garmin Drive51). Current velocity (V) was measured with a LJD‐10 velocity meter; water depth and river width were measured using a tape measure and rangefinder (LRB5000); dissolved oxygen (DO), pH, conductivity (Cond), turbidity (Turb), and water temperature (WT) were measured with a portable Yellow Springs Instrument (YSI6600, USA). Additionally, a 100 mL stream water sample was collected and brought back to the laboratory to measure the contents of silicate (SiO_2_‐Si), nitrate nitrogen (NO_3_‐N), ammonium nitrogen (NH_4_‐N), total nitrogen (TN), total phosphorus (TP), and phosphate (PO_4_‐P) following the standard analysis procedures (Chinese NEPA, 2002). The values of each environmental variable are shown in Table 2.

**TABLE 2:** Mean, standard deviation, and range of each environmental variable.

Environmental variables	Abbreviation	Mean	SD	Range
Elevation (m)	Elevation	2525.75	327.92	2000–3130
Water depth (m)	Dept	0.24	0.09	0.1–0.5
River width (m)	Width	9.98	10.59	0.6–37.5
Current velocity (m/s)	V	0.57	0.22	0–1.0
Total nitrogen (mg/L)	TN	0.27	0.08	0.09–0.48
Ammonium nitrogen (mg/L)	NH_4_‐N	0.017	0.01	0.003–0.061
Nitrate nitrogen (mg/L)	NO_3_‐N	0.23	0.08	0.07–0.48
Total phosphorus (mg/L)	TP	0.027	0.025	0.009–0.163
Phosphate (mg/L)	PO_4_‐P	0.023	0.025	0.009–0.163
Silicate (mg/L)	SiO_2_‐Si	3.28	0.81	2.15–6.38
Conductivity (μs/cm)	Cond	67.9	22.3	8.6–98.6
Dissolved oxygen (%)	Do	99.8	9.53	82.8–115.3
pH	pH	8.23	0.18	7.94–8.68
Water temperature (°C)	WT	8.2	1.5	4.7–10.7
Total dissolved solids (mg/L)	TDS	66.1	18.0	29.25–90.35
Turbidity (NTU)	Turb	16.1	31.1	1.96–203

Because diatoms can spread between sites via various means, MEM (Moran's Eigenvector Map) and AEM (Asymmetric Eigenvector Maps) were used to model the spatial dispersal of diatoms along geographical (overland dispersal) and network (directional watercourse) pathways between sample sites, respectively (Hu et al., 2022; Liu et al., 2013). MEM is a spatial variable analysis model that can carry out PCoA (Principal Co‐ordinates Analysis) of the geographical distance between any two sample sites, so it can model any undirected spatial structures from fine‐scale to broad‐scale (Borcard & Legendre, 2002). AEM is another spatial analysis model that is used to model directional spatial processes (Blanchet et al., 2011). Specifically, a binary matrix of sites × edges was established through the geographical coordinate of sites and the directional edges; then weights were assigned to each edge. Weight was calculated as Weight = 1 − (*d*/*d*_max_)^2^, where d is the watercourse distances between sites, and *d*_max_ is the maximum watercourse distances (Borcard et al., 2011). Geographical distances between sampling sites were calculated using latitude and longitude coordinates; the watercourse distances were calculated using the Network Analyst extension/OD Cost Matrix tool in ArcGIS 10.0. MEM was conducted based on geographical distances between sites using function *pcnm* in R package “vegan,” while AEM was computed using function *aem* in R package “AEM.”

### 2.5 Statistical analyses

Beta diversity partitioning based on the *Sørensen* index was used to calculate beta diversity and its components: total beta diversity (*β*_sor_), turnover (*β*_sim_), and nestedness (*β*_sne_). Taxonomic beta diversity (total, attached, and unattached communities) was calculated using the function *beta. pair* in package “betapart” (Baselga & Orme, 2012). For functional beta diversity, function *gowdis* in package “FD” was first used to generate the gowdis distance matrix of the 13 functional traits. Then, a Principal Coordinate Analysis (PCoA) was applied to produce trait vectors using function *cmdscale* from R package “labdsv” (Perez Rocha et al., 2019); the first three axes that explained 76.26% of the total variation were selected to represent all functional traits information (Villéger et al., 2013). Finally, the three trait vectors and taxonomic matrix were used to calculate functional beta diversity and its components using function *functional.beta.pair* in package “betapart.”

Mantel test was used to examine the relationship between taxonomic beta diversity and functional beta diversity. Mantel test was employed using *mantel* function in “vegan” package with 999 permutations. To test the relative contributions of environmental and spatial variables on diatom beta diversity, we employed distance‐based redundancy analysis (db‐RDA) followed by variation partitioning analysis (VPA). Firstly, we performed db‐RDA using the *rda* function and tested the significance using the *anova* function. Only if the test is significant can a forward selection be performed to identify important environmental and spatial variables that were correlated significantly with response matrices. Forward selection processes were carried out using the function *forward.sel* in “packfor” package and were based on two stopping criteria: the adjusted coefficient of determination ($R_{\mathrm{adj}}^{2}$) of the global model and significance level (*p* < .05). Finally, the selected factors were used as explanatory variables for the variation partitioning analysis using *varpart* in “vegan” package to calculate the unique and shared effects of environmental and spatial variables on diatom assemblages.

All data analyses were conducted in R (version 3.4.3. and 3.6.3).

## 3 RESULTS

### 3.1 The composition of species and functional traits of benthic diatom assemblages

**FIGURE 2:**
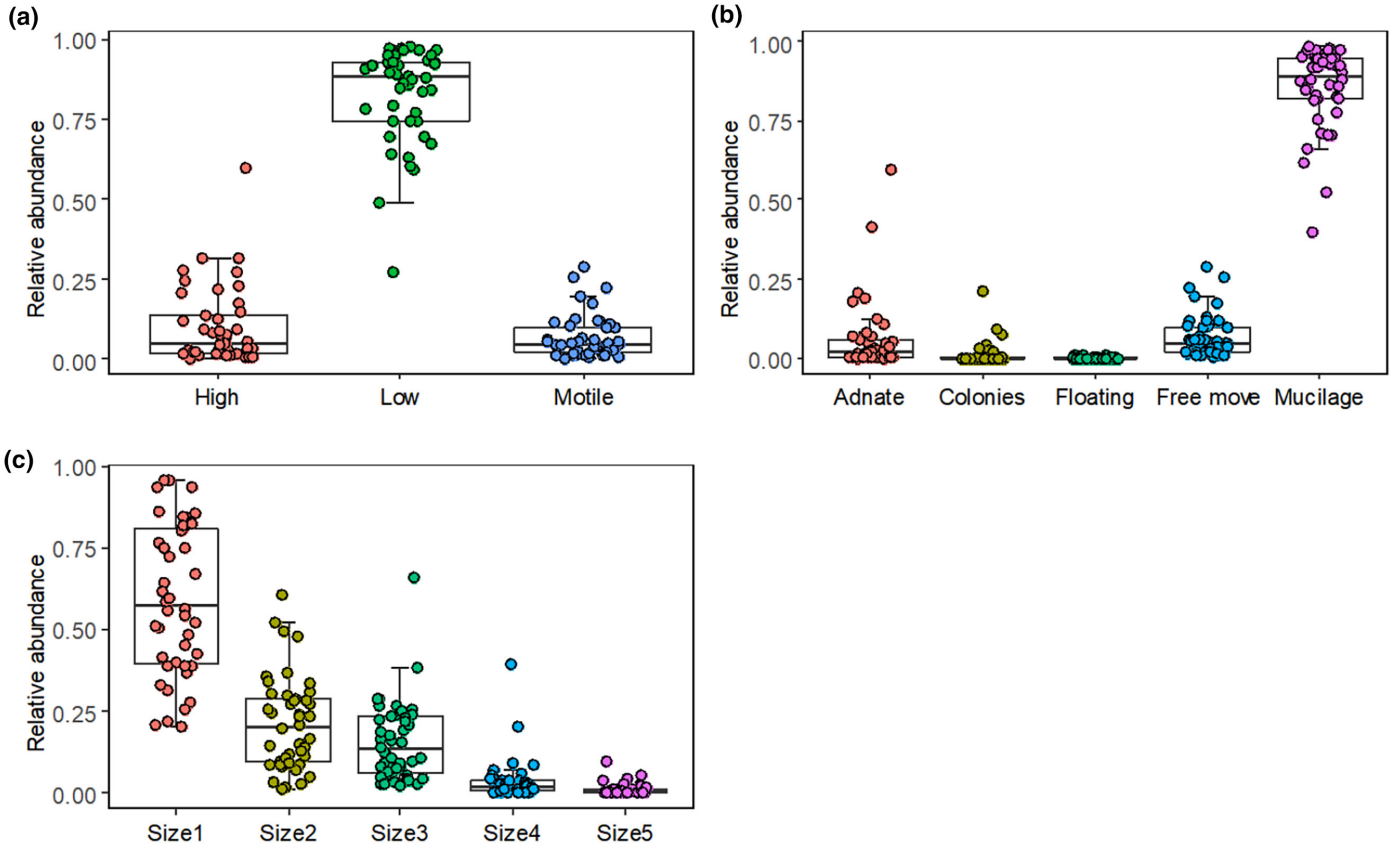
The relative abundance of functional traits for (a) guild profiles, (b) life forms, and (c) cell sizes distributed across diatom communities. Size1 < 100 μm^3^, 100 μm^3^ ≤ Size2 < 300 μm^3^, 300 μm^3^ ≤ Size3 < 600 μm^3^, 600 μm^3^ ≤ Size4 < 1500 μm^3^, Size5 ≥ 1500 μm^3^.

A total of 114 benthic diatom species were found in the Zhubaluo River, in which *Achnanthidium deflexum* (with a mean relative abundance of 54.8%), *Achnanthes suchlandtii* (6.6%), *Cocconeis neodiminuta* (5.6%), and *Cymbella sinuata* (5.1%) were dominant diatom taxa. For functional traits, the dominant ecological guild was the low‐profile guild (with a mean relative abundance of 83.0%), followed by the high‐profile (10.1%) and motile (6.9%) guilds (Figure 2a). The mean relative abundances of the life forms were distributed as follows: mucilage (85.8%), free move (6.9%), adnate (6.0%), colonies (1.2%), and floating (0.2%) (Figure 2b). On average, the cell sizes were distributed as follows: size1 (58.7%), size2 (21.1%), size3 (15.5%), size4 (3.5%), and size5 (1.2%) (Figure 2c).

### 3.2 The patterns and drivers of taxonomic and functional diatom beta diversity

**FIGURE 3:**
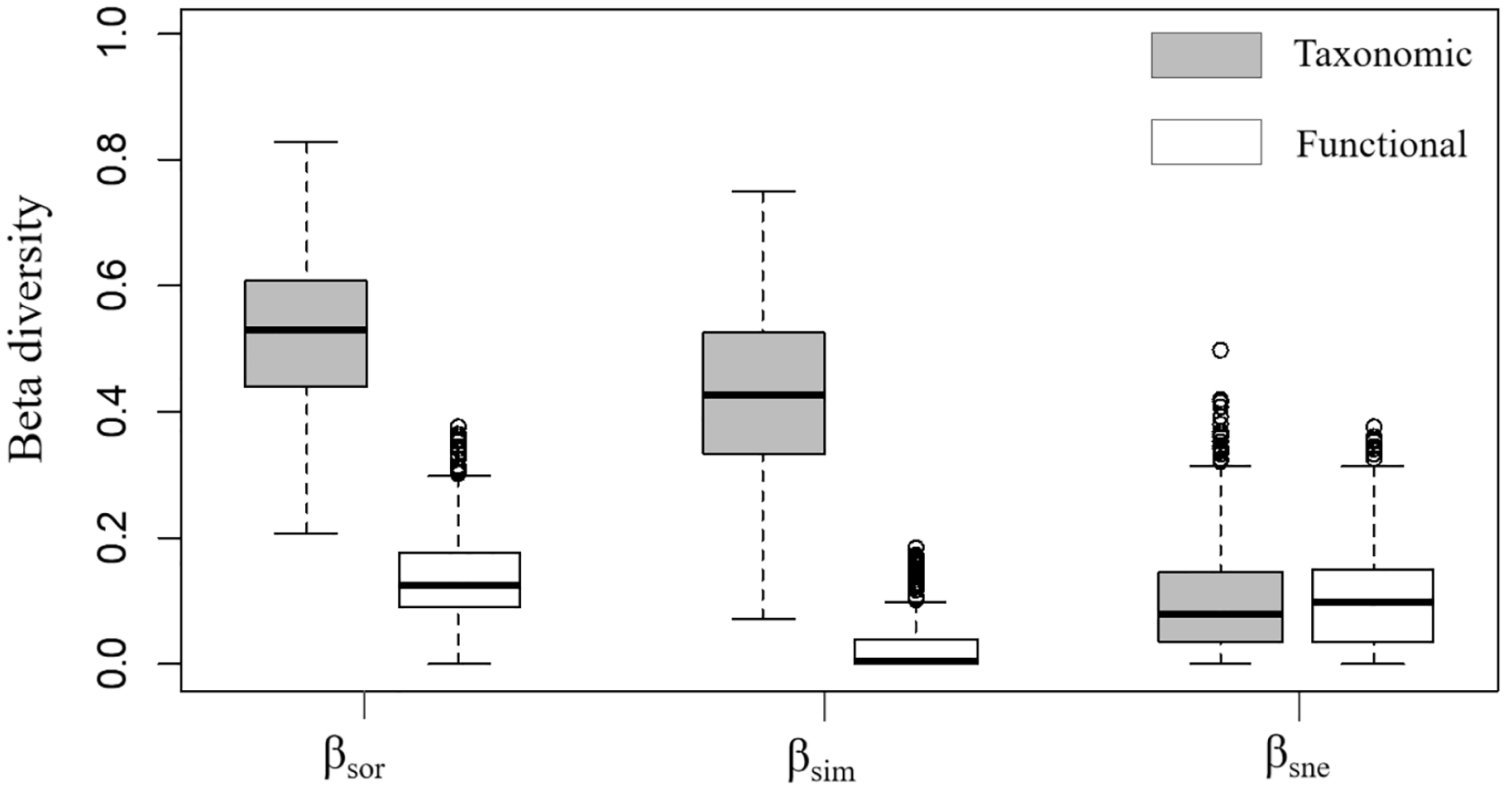
Boxplots of different components of pairwise dissimilarities for taxonomic and functional beta diversity. The bottom and top of the box are the 25th and 75th percentiles, and the band is the median. The gray box represents the taxonomic beta diversity, and the white box represents the functional beta diversity. *β*_sor_ = total beta diversity, *β*_sim_ = turnover beta diversity, *β*_sne_ = nestedness beta diversity.

**FIGURE 4:**
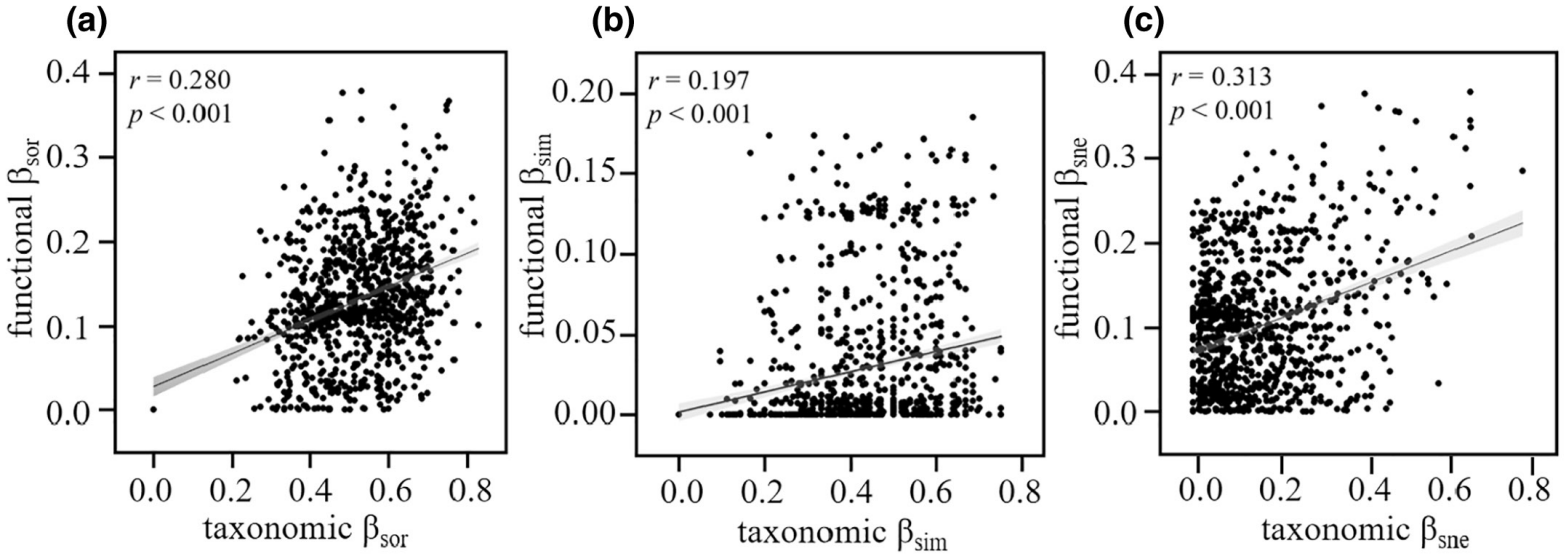
Relationship between taxonomic beta diversities and functional beta diversities, as measured with the (a) *Sørensen* dissimilarity (*β*_sor_) and its (b) turnover (*β*_sim_), and (c) nestedness (*β*_sne_) components. Mantel correlation coefficient (*r*) and significance (*p*) of each relationship are shown.

The mean of taxonomic beta diversity (*β*_sor_ = 0.527, *β*_sim_ = 0.424, and *β*_sne_ = 0.103) was greater than that of functional beta diversity (*β*_sor_ = 0.133, *β*_sim_ = 0.029, and *β*_sne_ = 0.104) (Figure 3). The patterns of taxonomic and functional beta diversity displayed differences, that is, total taxonomic beta diversity was mainly due to the turnover component (*β*_sim_ accounted for 80.5% of total dissimilarity), whereas total functional beta diversity was dominated by the nestedness component (*β*_sne_ accounted for 78.2% of total dissimilarity) (Figure 3). The Mantel test revealed a significant positive correlation between taxonomic beta diversities and functional beta diversities, indicating an association between dissimilarity in species composition and dissimilarity in functional traits (Figure 4).

Forward selection showed that mainly physical environmental variables (e.g., elevation, TDS, depth, and width) were selected for taxonomic beta diversity, whereas mainly chemical environmental variables (e.g., Cond, pH, and NH_4_‐N) were selected for functional beta diversity. Moreover, more spatial variables were selected for taxonomic beta diversity than functional beta diversity (Table 3).

**TABLE 3:** The selected variables according to the forward selection procedure.

	Env	MEM	AEM
Taxonomic			
*β*_sor_	Elevation, TDS, Depth, Width	MEM1, MEM2, MEM3, MEM4, MEM6	AEM1, AME2, AEM3, AEM4, AEM6, AEM14, AEM16, AEM25,AEM30
*β*_sim_	Elevation, TDS, Depth, Width	MEM1, MEM2, MEM3, MEM4, MEM6	AEM1, AEM2, AEM3, AEM4, AEM6, AEM14, AEM16, AEM25, AEM30
Functional			
*β*_sor_	Cond, WT, pH, NH_4_‐N	MEM1, MEM5	AEM1, AEM16
*β*_sne_	Cond, WT, pH, NH_4_‐N	MEM5	AEM1
Attached			
*β*_sor_	Cond, DO, Elevation, TDS, Depth, Width	MEM1, MEM2, MEM3, MEM4, MEM6	AEM1, AEM2, AEM4, AEM6, AEM14
*β*_sim_	Elevation, TDS	MEM1, MEM2, MEM3, MEM8	AEM6, AEM24, AEM28
Unattached			
*β*_sor_	Elevation, TDS, Width	MEM1, MEM2, MEM3, MEM8	AEM1, AEM2, AEM6, AEM24, AEM25
*β*_sim_	Elevation, TDS	MEM1, MEM2, MEM3, MEM8	AEM6, AEM24, AEM28
*β*_sne_	NH_4_‐N, Width	MEM1	AEM1, AEM2, AEM16, AEM25, AEM30

**FIGURE 5:**
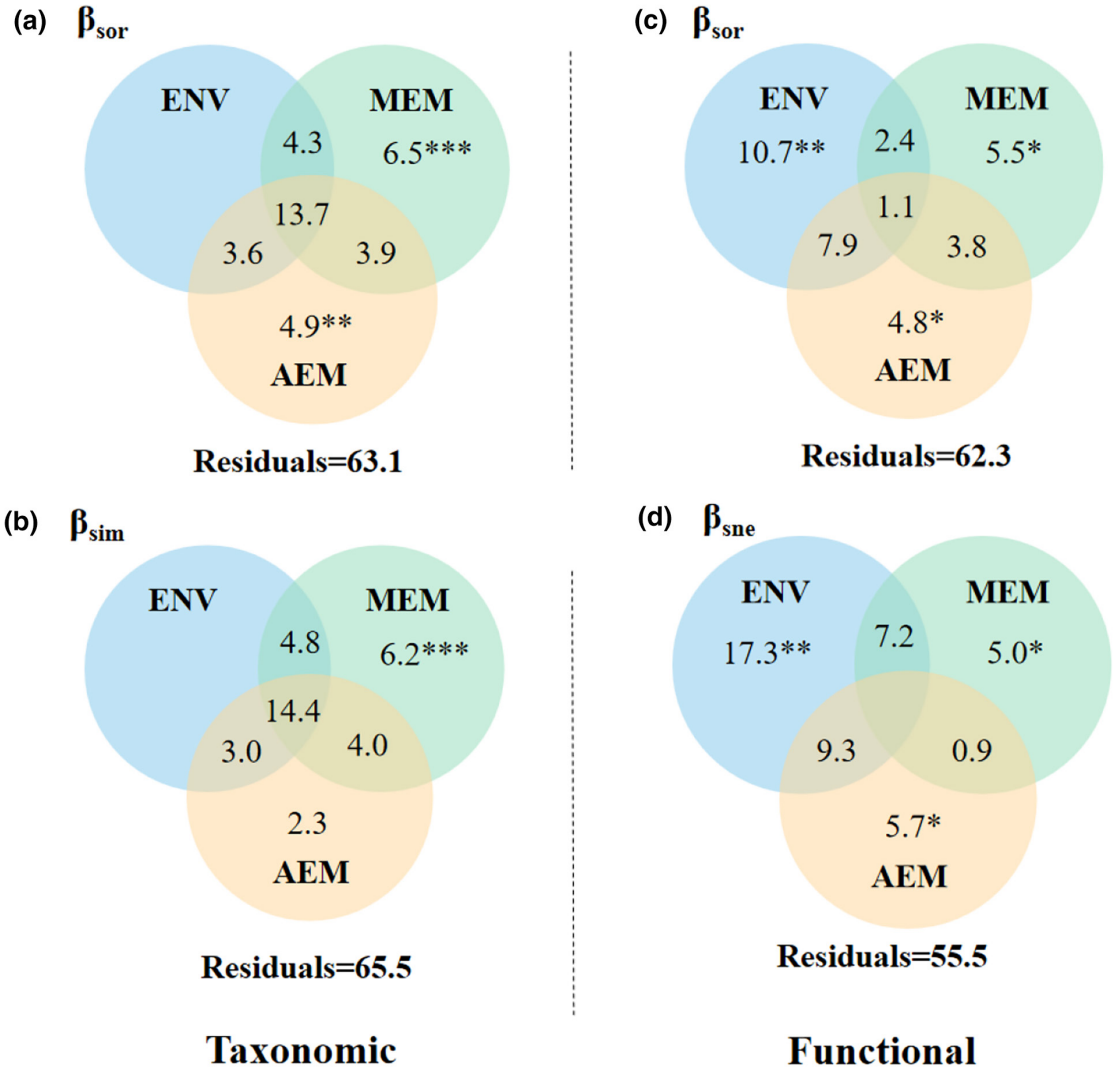
Venn diagrams show the relative contribution of selected environmental and spatial explanatory variables to taxonomic and functional beta diversity and its components. Values < 0 not shown. Values of Residuals represent the unexplained fractions. * indicate significance, *p < .05, **p < .01, ***p < .001. AEM, directional asymmetric eigenvector map models; Env, environmental explanatory variables; MEM, non‐directional Moran's eigenvector map models.

The results of variation partitioning analysis showed the different contributions of environmental and spatial explanatory variables to taxonomic and functional beta diversity and their components (Figure 5). For taxonomic beta diversity, environmental and spatial variables jointly explained the greatest amount of variation in taxonomic *β*_sor_ (13.7%) and *β*_sim_ (14.4%) (Figure 5a,b). In addition, unique spatial effects (explained 11.4% and 8.5% variation for *β*_sor_ and *β*_sim_, respectively) were more important than environmental effects (the contribution rate was negligible) in taxonomic beta diversity (Figure 5a,b). By comparison, the unique effect of environmental variables were dominant for functional beta diversity, which explained 10.7% and 17.3% variation for functional *β*_sor_ and *β*_sne_, respectively, whereas the pure contributions of spatial factors (ranging from 4.8% to 5.7%) were lower for functional beta diversity (Figure 5c,d). Moreover, variation partitioning was not computed for taxonomic *β*_sne_ and functional *β*_sim_ because no environmental and spatial explanatory variables were retained by forward selection.

### 3.3 The patterns and divers of attached and unattached diatom beta diversity

**FIGURE 6:**
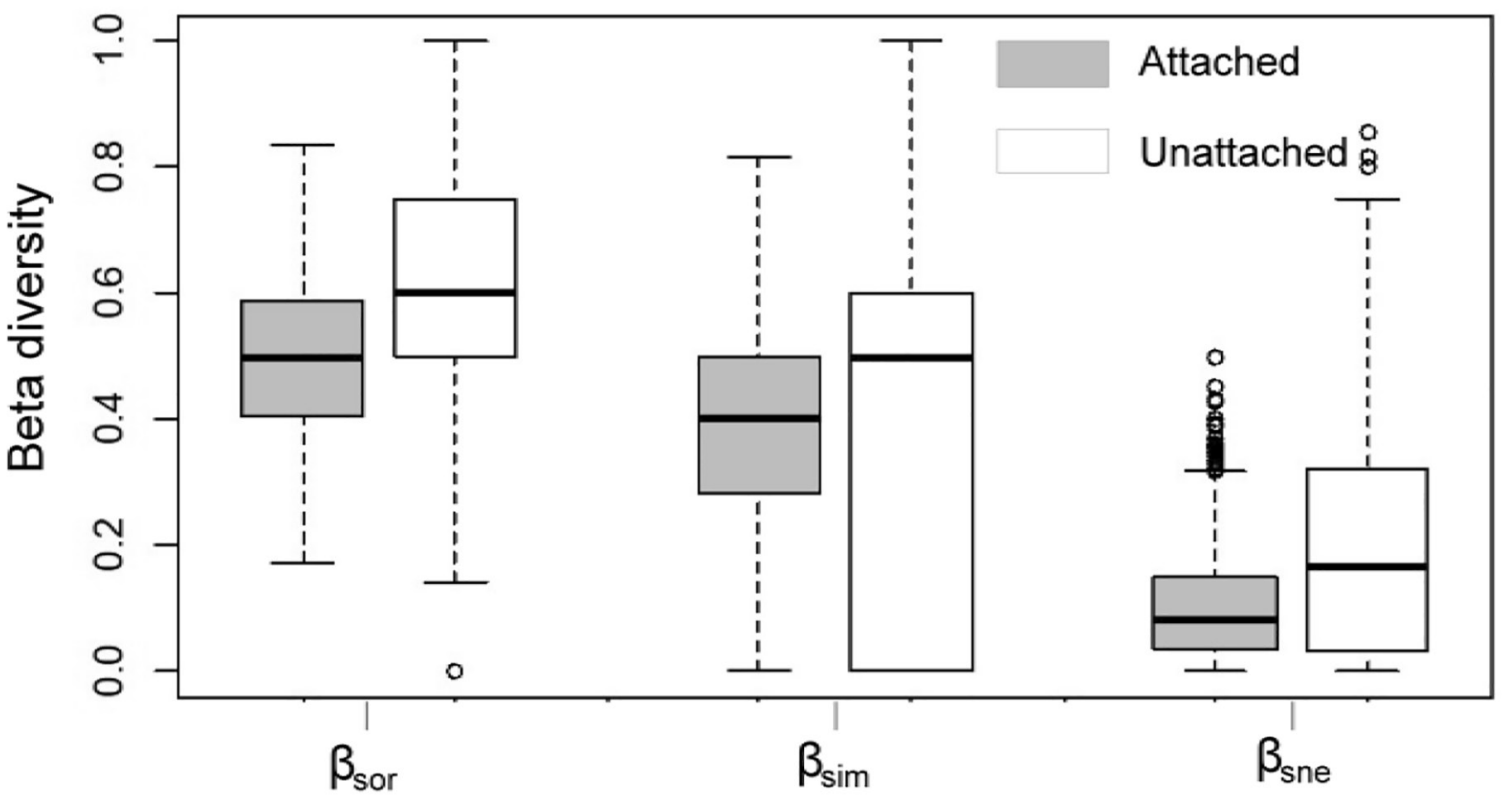
Boxplots of different components of pairwise dissimilarities for attached and unattached diatom beta diversity. The bottom and top of the box are the 25th and 75th percentiles, and the band is the median. The gray box represents the attached beta diversity, and the white box represents the unattached beta diversity. *β*_sor_ = total beta diversity, *β*_sim_ = turnover beta diversity, *β*_sne_ = nestedness beta diversity.

By dividing the diatom community into attached and unattached groups, it was found that the patterns of beta diversity of attached and unattached assemblages were both dominated by species turnover (Figure 6). Moreover, the mean beta diversity of unattached assemblages (*β*_sor_ = 0.61, *β*_sim_ = 0.41, and *β*_sne_ = 0.20) was greater than that of attached assemblages (*β*_sor_ = 0.49, *β*_sim_ = 0.39, and *β*_sne_ = 0.10) (Figure 6).

**FIGURE 7:**
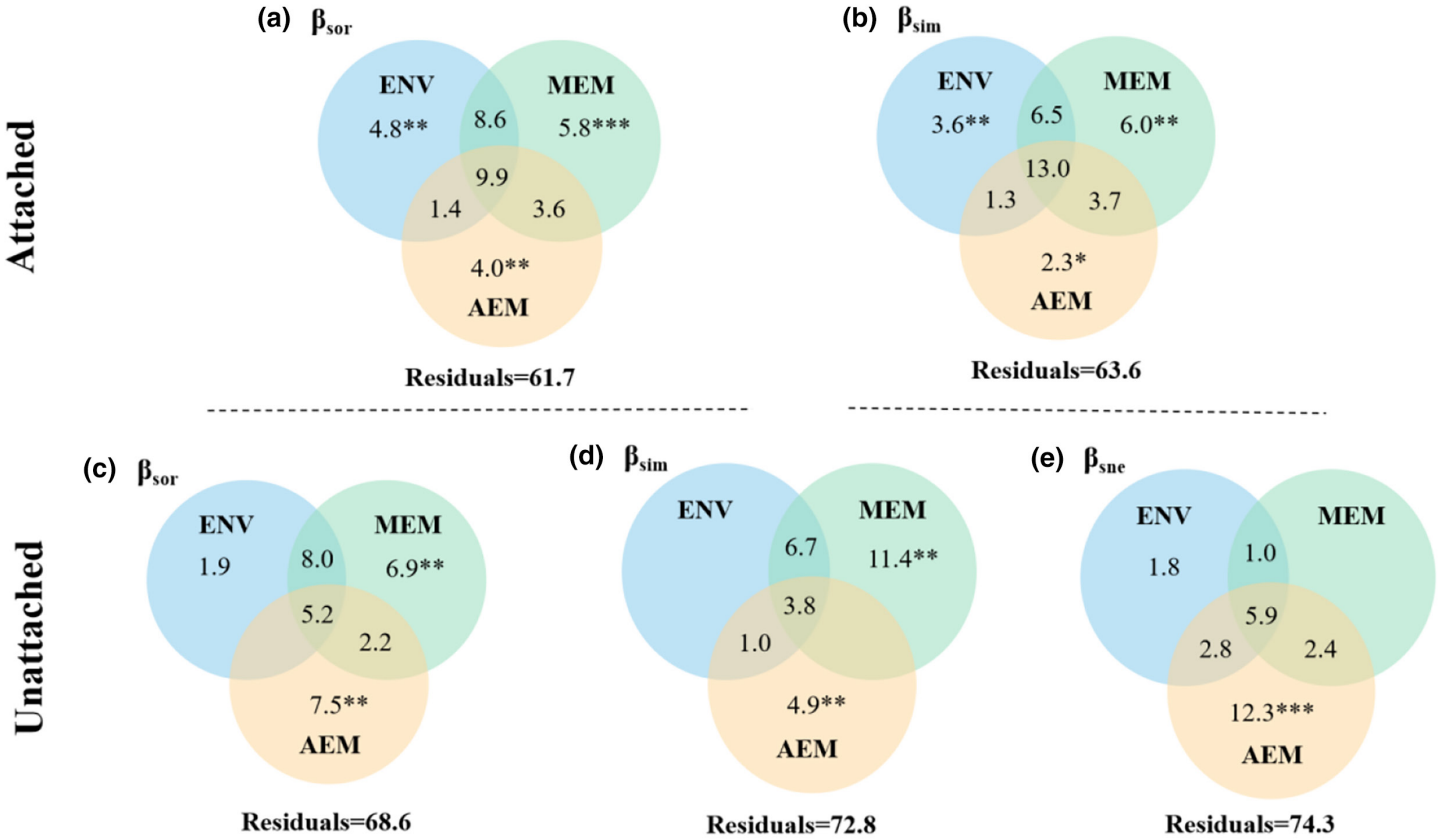
Venn diagrams show the relative contribution of selected environmental and spatial explanatory variables to attached and unattached diatom beta diversity and their components. Values <0 not shown. Values of Residuals represent the unexplained fractions. * indicate significance, *p < .05, **p < .01, ***p < .001. AEM, directional asymmetric eigenvector map models; Env, environmental explanatory variables; MEM, non‐directional Moran's eigenvector map models.

Variation partitioning analysis showed that both pure effects of environmental and spatial variables were important drivers shaping attached assemblages; yet, their joint effects were also considerable (Figure 7a,b). For unattached assemblages, unique spatial effects accounted for most variation, in which the contribution of AEM variables was generally higher than MEM variables in most beta components (Figure 7c–e). By comparison, environmental variables independently accounted for less variation (1.9%, 0%, and 1.8%) (Figure 7c–e).

## 4 DISCUSSION

### 4.1 The patterns of diatom beta diversity

By partitioning taxonomic and functional beta diversity, our results supported our first hypothesis that taxonomic beta diversity was primarily driven by turnover component, whereas functional beta diversity was mainly caused by nestedness component. Previous studies have observed similar results for stream algae in a large subarctic river (Perez Rocha et al., 2019) and temperate river (Wu et al., 2022). It is easy to understand that turnover component contributes more to taxonomic beta diversity in the Zhubaluo River, because this alpine stream has a large elevational gradient and fast flow that promotes high species turnover between sites. Indeed, species turnover has been reported to be the dominant component in most studies using beta diversity partitioning (Soininen et al., 2018). Compared with taxonomic beta diversity, we found that functional beta diversity displayed different patterns, with nestedness being the predominant component. One reason for this result may be the loss of some species with unique traits between sites due to environmental filtering, resulting in some traits are more common than others. This is supported by our finding that some traits such as low‐profile guild, mucilage, and small diatoms were dominate in Zhubaluo River. Therefore, some sites with low functional beta diversity become subsets of sites with high functional beta diversity (Perez Rocha et al., 2019; Wu et al., 2022). Another possible explanation is that, due to directional flow and geographical barrier in the Zhubaluo basin, habitat heterogeneity varies as large ranges from low to high across sites, resulting in few traits occurring in the low heterogeneity sites and a large variety of traits occurring in the high heterogeneity sites (Perez Rocha et al., 2019).

We also found that total taxonomic beta diversity was higher than that of total functional beta diversity. A high taxonomic beta diversity and low functional beta diversity were due to a high level of species turnover and a low functional turnover occurring in Zhubaluo River. This finding showed that while species composition varied greatly between sites, the variation of functional traits between sites might be much lower, as has been found in previous studies on various aquatic taxa, such as diatoms (Perez Rocha et al., 2019; Wu et al., 2022), macroinvertebrate (Heino & Tolonen, 2017), fishes (Villéger et al., 2013), and macrophyte (Fu et al., 2019). Despite the high species turnover rate among sites, there is a high level of functional convergence occurring, as different species within communities may exhibit similar functional strategies (Villéger et al., 2013). Thus, species replacements do not necessarily result in large changes in functional composition (Wu et al., 2022).

However, it is noteworthy that we found a significant correlation between taxonomic and functional beta components. This result suggested that changes in functional diversity were associated with species turnover or loss despite the weak correlation (Mantel correlation coefficients ranging from 0.197 to 0.313). This finding aligns with previous studies that have observed linkages between taxonomic and functional beta diversity, indicating that species replacement can drive community changes in functional level (Villéger et al., 2012; Wu et al., 2022). In particular, compared to the total and turnover components, we found that both taxonomic and functional nestedness components were relatively low, but they exhibited a stronger correlation. This result further indicated that the selective loss of species may be not random—it is preferentially eliminating species with unique functional traits (Si et al., 2016). This selective loss of functionally unique species drove the observed coupling between taxonomic and functional nestedness. For instance, one could imagine that certain poorly attached diatoms would disappear at some sites due to rapid flow, resulting in taxonomic and functional nestedness components. Overall, the weak correlation demonstrated the associations and differences between species and functional composition while also providing complementary information in understanding diatom beta diversity from different facets.

In addition, the different patterns observed in species and functional beta diversity have provided valuable insights for biodiversity conservation. Several reports have demonstrated that turnover implies multiple sites need to be protected, while nestedness suggested that conservation should target the richest sites (Peláez & Pavanelli, 2019; Socolar et al., 2016). Our results suggested that emphasis should be placed on the protection of multiple sites with different species combinations and richer sites with higher functional diversity. This is because there is substantial species replacement between different sites, and protecting a single site alone would not capture the whole regional biodiversity (Baselga, 2010). Similarly, the sites with higher functional nestedness components contain the majority of functional traits found in other sites, and are key to maintaining the regional biodiversity. This comprehensive approach, targeting both taxonomic and functional beta diversity, represents an effective means of conserving the regional biodiversity (Villéger et al., 2013; Wu et al., 2022).

### 4.2 The drivers of diatom beta diversity

Our results confirmed the second hypothesis that the responses of functional beta diversity to environmental processes were more significant, whereas taxonomic beta diversity was rather more affected by spatial patterns. This is because changes in functional diversity can capture important signals related to environmental filtering that may not be evident from patterns of taxonomic diversity (Siefert et al., 2013; Swenson, 2011). This occurs as different species can display similar functional traits over broad temporal or spatial scales (Peláez & Pavanelli, 2019). Consequently, some reports have recommended that traits‐based rather than taxa‐based diatom indicators are more suitable for river biomonitoring (B‐Béres et al., 2016; Wu et al., 2017). Several studies have found spatial patterns of diatom communities on different temporal and spatial scales (Bottin et al., 2014; Falasco et al., 2019; Potapova & Charles, 2002; Soininen, 2007; Tang, Wu, et al., 2013), but these results may be worth discussing when considering the variations in functional compositions. Recent studies indicated that the patterns and drivers of beta diversity were scale dependent (He et al., 2023; Soininen, 2023), but how this dependency exists in functional assemblages is not clear. Therefore, analyzing the drivers of community assembly relying solely on species diversity may not be sufficient without considering the functional facet of biodiversity (Swenson, 2011; Villéger et al., 2013).

Our results also showed that spatial and environmental processes formed beta diversity patterns of diatom assemblages through species turnover and functional nestedness. Species turnover describes species replacement among sites, the potential drivers include environmental filtering, species interaction, geographical isolation (Legendre, 2014). We found that species turnover was mainly driven by spatially structured environmental variables and non‐directional spatial processes (i.e., MEM). The finding implied that spatial processes appeared as the primary determinant of community assembly, resulting in species replacement among sites. Specifically, dispersal limitation caused by geographical barriers can result in dissimilarity in species composition among highly isolated locations (Peláez & Pavanelli, 2019). Moreover, environmental processes can serve as a filter, permitting species suited to the local environment to settle (Heino, Melo, Siqueira, et al., 2015). It is just that the environmental processes were covariant along geographical space, so some unique environmental processes were masked. Previous studies also reported such spatially structured environmental processes and suggested they were inextricable and unavoidable (Hu et al., 2022; Vilmi et al., 2016).

By comparison, functional nestedness was driven primarily by environmental processes, which may be associated with selective extinction (Heino & Tolonen, 2017; Si et al., 2016). Alpine streams are highly heterogeneous, encompassing large environmental variations like fast flow, temperature, and ion concentrations (Falasco et al., 2019). This heterogeneity may cause some species sensitive to environmental changes to disappear (selective extinction) (Wu et al., 2022). This conjecture may be supported by the result that diatom traits with low environmental tolerance (e.g., high profile) and high dispersal ability (e.g., motile profile, floating) accounted for a small proportion. In addition, we found that spatial processes also played an essential role in shaping functional nestedness. The interaction between dispersal limitation and directional flow likely resulted in low turnover and high nestedness components of functional traits (Heino et al., 2017). We thus infer that certain traits with low environmental tolerance and high dispersal ability disappear due to environmental filtering and directional flow, resulting in low functional diversity assemblages becoming subsets of the high functional diversity assemblages. Therefore, the patterns of turnover and nestedness depend on the interaction between environmental heterogeneity and dispersal limitation: heterogeneity environment and dispersal limitation may lead to a higher functional nestedness (Gianuca et al., 2017; Peláez & Pavanelli, 2019).

### 4.3 Division of benthic diatom community

When compared with the total community, we found that the beta diversity patterns of attached and unattached assemblages were also based on species turnover, implying that species replacement was the main driver of taxonomic beta diversity. We also found that both environmental and spatial variables were important drivers shaping diatom assemblages; yet, their relative importance varied considering different attached abilities. Environmental and spatial variables were important drivers for attached assemblages, whereas unattached assemblages showed stronger spatial patterns, which is basically consistent with our third hypothesis.

Attached diatoms can respond well to local environmental changes because they occupy stable habitats while also being influenced by dispersal limitation resulting from geographical barriers (Bottin et al., 2014; Dong et al., 2016). Therefore, the attached assemblages showed turnover patterns under the multiple effects of environmental filtering, directional flow, and dispersal limitation. In the whole community, however, the responses of diatoms to environmental processes appeared to be overwhelmed, and spatial processes dominated instead. In comparison, we found that unattached diatoms were strongly influenced by stochastic processes, especially directional spatial processes (i.e., AEM), whereas the effects of environmental processes were negligible. It is likely that directional flow promoted species replacement among assemblages (Pozzobom et al., 2021). However, we found that non‐directional spatial processes were also important for turnover patterns of unattached diatoms. This can be explained by the fact that the flow is unidirectional in the stream network (Campbell Grant et al., 2007; Peterson et al., 2013), so dispersal limitation remains (e.g., dispersal from downstream to upstream) (Srivastava & Kratina, 2013). A noteworthy result is that the nestedness components of unattached diatoms were strongly affected by directional spatial processes. It seems that the directional flow caused the rapid disappearance of some unattached diatoms (such as motile profile and floating) in a certain habitat, resulting in partly functional nestedness structures. Overall, these results showed that dividing the whole community can identify potential driving processes, which deepened our understanding of the patterns and drivers of beta diversities and their components, especially in the alpine streams.

## 5 CONCLUSIONS

In conclusion, our study revealed that the beta diversity patterns of benthic diatoms in Alpine streams were dominated by species turnover and functional nestedness. Dispersal limitation and directional flow shaped the spatial patterns of diatom communities and promoted species replacement among assemblages, as species are treated as functionally equivalent. However, traits‐based diatom assemblages can well capture the signal of environmental filtering and form nestedness patterns under the effects of both environmental and spatial processes. Furthermore, we clarified the potential causes for forming this diversity pattern by dividing the whole communities based on the attached ability, an essential adaptive strategy of benthic diatoms in alpine streams. We found that the varied responses of benthic diatoms with different attached abilities to environmental filtering, dispersal limitation, and directional flow facilitated species turnover and functional nestedness in the alpine stream. Therefore, our study provides valuable insight into community assembly processes and biodiversity conservation by exploring different aspects (taxonomic and functional) and components (turnover and nestedness) of beta diversity. Additionally, our study highlighted the importance of deconstructing the whole biotic community based on crucial adaptive strategies in understanding multiple metacommunity processes.

## AUTHOR CONTRIBUTIONS

**Jiancheng Hu:** Conceptualization (equal); data curation (lead); investigation (lead); methodology (lead); software (lead); visualization (lead); writing – original draft (lead); writing – review and editing (equal). **Nuo Xu:** Data curation (equal); visualization (supporting); writing – original draft (supporting); writing – review and editing (equal). **Sicheng Ao:** Data curation (supporting); investigation (equal); writing – review and editing (supporting). **Lu Tan:** Data curation (equal); investigation (equal); project administration (supporting). **Xianfu Li:** Data curation (supporting); investigation (equal). **Qinghua Cai:** Conceptualization (supporting); funding acquisition (supporting); investigation (supporting); project administration (equal). **Tao Tang:** Conceptualization (lead); data curation (equal); funding acquisition (lead); methodology (equal); project administration (lead); writing – original draft (supporting); writing – review and editing (equal).

## FUNDING INFORMATION

This research is supported by the National Natural Science Foundation of China (No. 32071589), Foundation of Talent Introduction Project of Hubei Polytechnic University (No. 23xjz06R), Hubei Provincial Natural Science Foundation and Huangshi of China (No. 2022CFD058) and the Young Scientists Fund of the National Natural Science Foundation of China (No. 32301334).

## CONFLICT OF INTEREST STATEMENT

The authors declare that there are no conflicts of interest.

## Supporting information


Data S1.

## Data Availability

The data that supports the findings of this study are available in Dryad at https://datadryad.org/stash/share/vni3FmhrE_ZO5Tw5AS1H09dUbTUi_kk8EHDS0k3jjRg.
